# *Med23* deficiency reprograms the tumor microenvironment to promote lung tumorigenesis

**DOI:** 10.1038/s41416-023-02556-9

**Published:** 2024-01-09

**Authors:** Xiaobo Fu, Siming Liu, Dan Cao, Chonghui Li, Hongbin Ji, Gang Wang

**Affiliations:** 1 State Key Laboratory of Genetic Engineering, School of Life Sciences and Zhongshan Hospital, Fudan University, Shanghai, 200438 China; 2 Shanghai Institute of Immunology, Department of Immunology and Microbiology, Shanghai Jiao Tong University School of Medicine, Shanghai, China; 3 State Key Laboratory of Cell Biology, Center for Excellence in Molecular Cell Science, Shanghai Institute of Biochemistry and Cell Biology, Chinese Academy of Sciences, University of Chinese Academy of Sciences, Shanghai, 200031 China

**Keywords:** Non-small-cell lung cancer, Cancer microenvironment, Mechanisms of disease

## Abstract

**Background:**

Lung cancer is the leading cause of cancer-related death worldwide. We previously found that Mediator complex subunit 23 (MED23) is important for the tumourigenicity of lung cancer cells with hyperactive Ras activity in vitro, although the in vivo function of MED23 in lung tumourigenesis remains to be explored.

**Methods:**

In this study, we utilized well-characterized *Kras*^G12D^-driven non-small cell lung cancer mouse model to investigate the role of MED23 in lung cancer. The lung tumour progression was evaluated by H&E and IHC analysis. Western blotting and qRT-PCR assays were performed to detect changes in gene expression. Immune cells were analyzed by FACS technology. RNA-seq and reporter assays were conducted to explore the mechanism.

**Results:**

We observed that lung epithelial *Med23* deletion by adeno-Cre resulted in a significant increase in *Kras*^G12D^ tumour number and size, which was further verified with another mouse model with *Med23* specifically deleted in alveolar type II cells. Mice with lung-specific *Med23* deficiency also exhibited accelerated tumourigenesis, and a higher proliferation rate for tumour cells, along with increased ERK phosphorylation. Notably, the numbers of infiltrating CD4^+^ T cells and CD8^+^ T cells were significantly reduced in the lungs of *Med23*-deficient mice, while the numbers of myeloid-derived suppressor cells (MDSCs) and Treg cells were significantly increased, suggesting the enhanced immune escape capability of the *Med23*-deficient lung tumours. Transcriptomic analysis revealed that the downregulated genes in Med23-deficient lung tumour tissues were associated with the immune response. Specifically, *Med23* deficiency may compromise the MHC-I complex formation, partially through down-regulating *B2m* expression.

**Conclusions:**

Collectively, these findings revealed that MED23 may negatively regulate Kras-induced lung tumourigenesis in vivo, which would improve the precise classification of *KRAS*-mutant lung cancer patients and provide new insights for clinical interventions.

## Introduction

Lung cancer is the leading cause of cancer-related death worldwide, with a 5-year survival rate of less than 18% [1]. Lung adenocarcinoma, the most common form of lung cancer (∼40%), is often diagnosed at an advanced stage and has a poor prognosis [2]. Over the last decade, the identification of driver mutations and fusions and development of specific targeted therapies, including tyrosine kinase inhibitors for EGFR mutations and crizotinib for ALK, RET, and ROS1 fusions, have significantly improved the overall survival of a few subsets lung cancer patients [3]. In addition, recent advancements in immunotherapies targeting immune checkpoint molecules, such as PD-1 and CTLA-4, have achieved remarkable success [4]. However, due to the susceptibility of patients to develop drug resistance and experience relapse, as well as the ongoing evolution of tumor cells leading to immune evasion, targeted therapies and immunotherapies have shown suboptimal therapeutic efficacy in some patients, especially for patients with KRAS mutation, who account for almost 30% of lung adenocarcinoma cases [5]. Even though novel advances in technology and approaches have facilitated the process of drug discovery, direct inhibition of mutant *KRAS* is still very challenging. Although there are a few different properties among different subtypes of KRAS mutations, all of these mutations lead to constitutive activation of downstream pathways, such as the MAPK pathway and PI3K pathway [6, 7]. Thus, targeting these effector pathways has been considered as a therapeutic alternative [8, 9].

The Mediator Complex (Mediator) is an evolutionarily conserved multiprotein complex that functions as an integrative hub regulating multiple pathways during development and diseases [10, 11]. Notably, an increasing number of mutations in many Mediator subunits or alterations in their expression have been reported in human cancers, indicating a crucial role for Mediator in tumorigenesis [12, 13]. MED23, a subunit in the tail module of Mediator, was originally identified as an important downstream regulator of the MAPK signaling pathway [14]. Recent research on MED23 revealed its critical role in diverse physiological processes, including adipogenesis, bone development, DNA damage repair and liver fibrosis [15–18]. We previously demonstrated that MED23 and its binding partner ELK1 were selectively important for the proliferation and tumorigenicity of lung cancer cell lines with *RAS* mutations [19]. However, whether MED23 functions during lung tumorigenesis in vivo remains to be investigated.

In this study, we utilized well-characterized models of lung adenocarcinoma that recapitulate human lung cancer through the activation of a *Kras*^G12D^ allele to investigate the role of MED23 in lung cancer. To our surprise, we found that deletion of *Med23* along with activation of *Kras*^G12D^ markedly accelerated lung tumorigenesis. By affecting tumor progression and proliferation, *Med23* deficiency in *Kras*^G12D^ mice resulted in an increasing number of tumors in the lungs. Mechanistically, *Med23* deletion altered the immune microenvironment. We also found significant changes in the percentages of infiltrating CD4^+^ T cells, CD8^+^ T cells and myeloid-derived suppressor cells (MDSCs) in the lungs of these mice, indicating an enhanced immune escape capability for the *Med23*-deficient lung tumors. Taken together, our data indicate that MED23 may also act as a tumor suppressor in *Kras*-induced lung cancer in vivo.

## Results

### *Med23* deletion promotes *Kras*-induced lung tumorigenesis in vivo

**Fig. 1:**
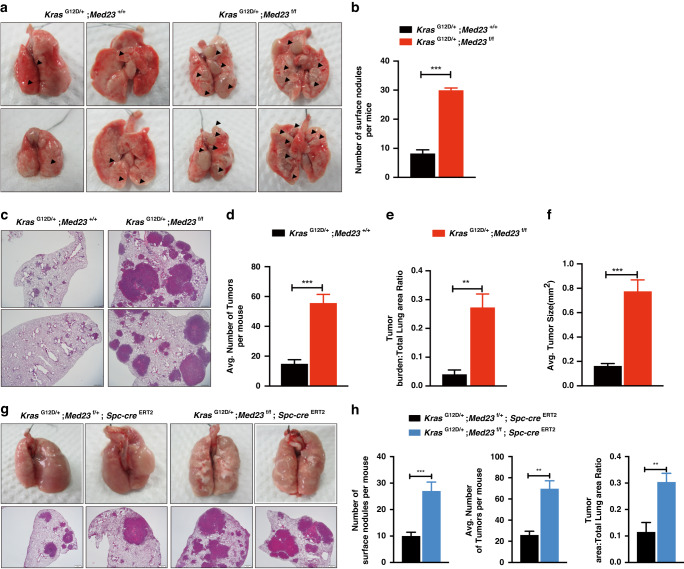
*Med23* deletion promotes *Kras*-induced lung tumorigenesis in vivo. **a** Representative views of surface nodules on the lungs of *Kras*^G12D/+^;*Med23*^+/+^ mice and *Kras*^G12D/+^;*Med23*^f/f^ mice after 16 weeks after Adeno Cre infection. Arrows point to the surface nodules. **b** Quantification of the surface nodules of *Kras*^G12D/+^;*Med23*^+/+^ mice and *Kras*^G12D/+^;*Med23*^f/f^ mice (*Kras*^G12D/+^;*Med23*^+/+^, n = 7; *Kras*^G12D/+^;*Med23*^f/f^, *n* = 8). **c** Representative H&E staining of lung sections from *Kras*^G12D/+^;*Med23*^+/+^ mice and *Kras*^G12D/+^;*Med23*^f/f^ mice 16 weeks after Adeno Cre infection. **d**–**f** Quantification of the tumor number and tumor size of *Kras*^G12D/+^;*Med23*^+/+^ mice and *Kras*^G12D/+^;*Med23*^f/f^ mice (*Kras*^G12D/+^;*Med23*^+/+^, *n* = 7; *Kras*^G12D/+^;*Med23*^f/f^, *n* = 8). **g** Histological analysis of lung tumors in *Kras*^G12D/+^;*Med23*^f/+^;*Spc-cre*^ERT2^ mice and *Kras*^G12D/+^;*Med23*^f/f^;*Spc-cre*^ERT2^ mice at 25 weeks after tamoxifen injection. **h** Quantification of the surface nodules, tumor numbers and tumor areas of *Kras*^G12D/+^;*Med23*^f/+^;*Spc-cre*^ERT2^ mice and *Kras*^G12D/+^;*Med23*^f/f^;*Spc-cre*^ERT2^ mice (*n* = 7 per group). Data are presented as the means ± SEMs. **P < 0.01, ***P < 0.001.

To investigate the role of *Med23* in lung cancer, we constructed a conditional *Med23*-knockout mouse model (*Med23*^f/f^), in which *Med23* deletion in a small percentage of pulmonary cells was achieved by intranasal delivery of adenoviral Cre recombinase (Adeno Cre) when the mice were 6–8 weeks old. By 25 weeks post-Adeno Cre infection, no lesions or inflammation were observed in the *Med23*^f/f^ mice, suggesting that *Med23* deletion alone seems not to form lung tumorigenesis by 25 weeks post-Adeno Cre infection (Fig. S1A). Our previous study showed that MED23 was required for *Ras*-driven lung cancer in vitro [19]. To further investigate the role of *Med23* in *Kras*-induced lung tumorigenesis in vivo, we crossed *LSL-Kras*^G12D/+^ mice with *Med23*^f/f^ mice. Activation of *Kras*^G12D^ with or without simultaneous deletion of *Med23* in lung epithelial cells was achieved via inhaled intranasal delivery of Adeno Cre to *LSL-Kras*^G12D/+^;*Med23*^+/+^ mice (hereafter referred to *Kras*^G12D/+^;*Med23*^+/+^ mice) and *LSL-Kras*^G12D/+^;*Med23*^f/f^ mice (hereafter referred to *Kras*^G12D/+^;*Med23*^f/f^ mice). Intuitively, based on previous in vitro results, we expected that *Med23* deletion would inhibit lung tumorigenesis in vivo. However, at 16 weeks post-Adeno Cre infection, *Kras*^G12D/+^;*Med23*^f/f^ mice showed dramatically more and larger tumor nodules on the lung surface than *Kras*^G12D/+^;*Med23*^+/+^ mice (Fig. 1a, b). Quantification of lung sections showed that there was an approximately 3.7-fold increase in tumor number in *Kras*^G12D/+^;*Med23*^f/f^ mice compared with control mice (Fig. 1c, d). Additionally, the tumors in *Kras*^G12D/+^;*Med23*^f/f^ mice were significantly larger (Fig. 1c, e, f; Fig. S1B). Notably, there was no impact on tumorigenesis when one *Med23* allele remained intact (*Kras*^G12D/+^;*Med23*^f/+^ mice) (Fig. S1C, D).

Adeno Cre infection can cause inflammation, and the infected cell types are complex. To further verify the phenotypes we found in *Kras*^G12D/+^;*Med23*^f/f^ mice, we used the *Spc-Cre*^ERT2^ mouse model, which allowed specific deletion of *Med23* in type II alveolar epithelial cells upon tamoxifen induction [20]. Compared with *Kras*^G12D/+^;*Med23*^f/+^;*Spc-cre*^ERT2^ mice, *Kras*^G12D/+^;*Med23*^f/f^;*Spc-cre*^ERT2^ mice showed a dramatic increase in the number of lung surface nodules at 25 weeks post intraperitoneal tamoxifen injection (Fig. 1g). Further quantification of lung sections showed that there was an ~2.7-fold increase in the tumor number and an ~2.65-fold increase in tumor area in *Kras*^G12D/+^;*Med23*^f/f^;*Spc-cre*^ERT2^ mice compared with control mice (Fig. 1h). Thus, these phenotypes suggested that deletion of *Med23* specifically in type II alveolar epithelial cells could also accelerate lung tumorigenesis, which was highly consistent with what we observed in the *Kras*^G12D/+^;*Med23*^f/f^ mice. Collectively, these results implied that genetic ablation of *Med23* in vivo greatly facilitated the *Kras*-induced lung tumorigenesis.

### *Med23* deficiency does not affect histological classification of tumors

**Fig. 2:**
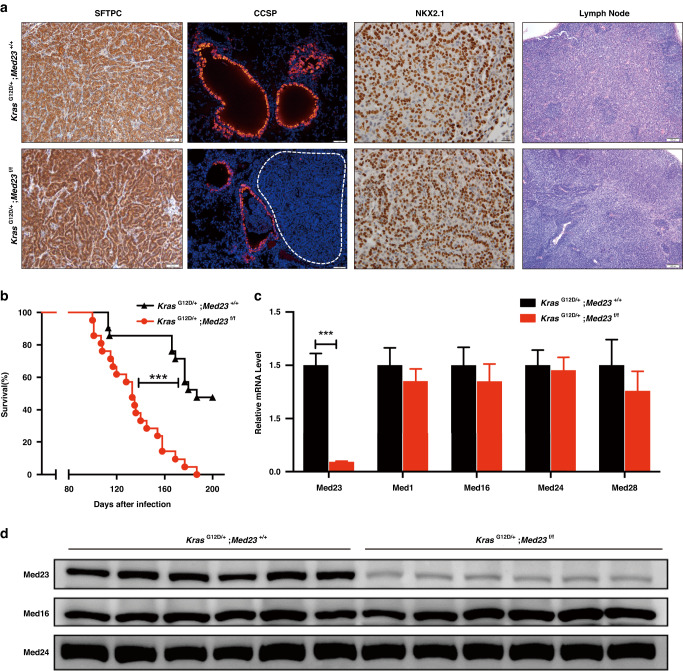
*Med23* deficiency does not affect histological classification of tumors. **a** Lung sections from *Kras*^G12D/+^;*Med23*^+/+^ mice and *Kras*^G12D/+^;*Med23*^f/f^ mice at 16 weeks post Adeno Cre infection were stained for SFTPC, CCSP, or NKX2.1. Lymph node sections from both groups were stained with H&E. **b** Kaplan‒Meier survival curves for *Kras*^G12D/+^;*Med23*^+/+^ mice and *Kras*^G12D/+^;*Med23*^f/f^ mice (*n* = 15 per group). **c** Total RNA was extracted from lung tumors from both groups at 20 weeks post-infection and then analyzed by qRT‒PCR to detect the mRNA levels of *Med23*, *Med1*, *Med16*, *Med24* and *Med28* (*n* = 7 per group). **d** Total protein was extracted from lung tumors from both groups at 25 weeks post-infection and then analyzed by Western blotting to detect the protein levels of MED23, MED16 and MED24. MED16 and MED24 were used as loading control for MED23. *n* = 3 mice per group, and two separate tumors were taken from each mice for Western blot analysis. Data are presented as the means ± SEMs. ***P < 0.001.

As there were huge changes in tumor number and size after *Med23* deletion, we sought to analyze the impact of *Med23* on the histological classification of tumors by staining. Both groups stained positive for NK2 homeobox 1 (NKX2.1), indicating that they were adenocarcinomas (Fig. 2a). In addition, tumors in both groups were positive for surfactant protein C (SFTPC) but negative for Clara cell secretory protein (CCSP) (Fig. 2a), suggesting that the tumors arose from alveolar cells. These results showed that *Med23* deficiency did not appear to affect cell origin or histological classification of tumors. In addition, at this time point, lymphatic metastasis was not observed in the mice with or without *Med23* deletion (Fig. 2a). However, as the *Kras*^G12D/+^ driven lung tumor model is not a metastatic model, whether *Med23* deficiency affects lung tumor metastasis is unclear and requires further study. Next, to investigate whether *Med23* deletion affects survival, a cohort of mice from both groups was treated with a higher titer of Adeno Cre. Compared with *Kras*^G12D/+^;*Med23*^+/+^mice, which had a median survival time of 187 days post-Adeno Cre infection, *Kras*^G12D/+^;*Med23*^f/f^ mice survived a markedly shortener time of only 133 days (Fig. 2b).

We also confirmed the deletion efficiency of *Med23* in *Kras*^G12D/+^;*Med23*^f/f^ mice. Compared with tumors in *Kras*^G12D/+^;*Med23*^+/+^ mice, those in *Kras*^G12D/+^;*Med23*^f/f^ mice showed great reductions in the mRNA and protein levels of *Med23* (Fig. 2c, d), suggesting a good recombination efficiency in our mouse model. Mediator is a multiprotein complex. To exclude the possibility of nonspecific effects on other Mediator components, the expression levels of a few other Mediator subunits were checked. As expected, no significant changes were observed in other Mediator subunits, including *Med16* and *Med24* (Fig. 2c, d), which were reported to interact with MED23 to form the tail subcomplex [21, 22]. Taken together, these data suggested that *Med23* deletion combined with *Kras*-induction promoted tumorigenesis in this in vivo animal model.

### *Med23* deletion promotes tumor progression and cell proliferation

**Fig. 3:**
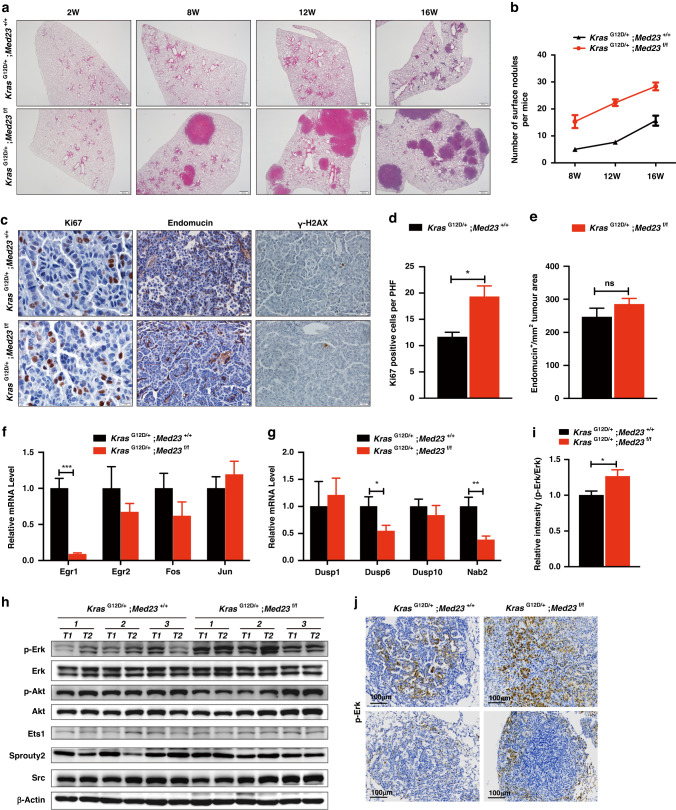
*Med23* deficiency promotes tumor progression and cell proliferation. **a** Representative H&E staining of lung sections from *Kras*^G12D/+^;*Med23*^+/+^ mice and *Kras*^G12D/+^;*Med23*^f/f^ mice collected at the indicated times post-infection. **b** Quantification of the surface nodules of *Kras*^G12D/+^;*Med23*^+/+^ mice and *Kras*^G12D/+^;*Med23*^f/f^ mice at the indicated times (*n* = 3 per group). **c** Lung sections from *Kras*^G12D/+^;*Med23*^+/+^ mice and *Kras*^G12D/+^;*Med23*^f/f^ mice at 16 weeks post Adeno Cre infection were stained for Ki67, Endomucin or γ-H2AX, and representative views are shown. **d,** **e** Quantification of Ki67-positive cells and endomucin-positive areas in lung sections from *Kras*^G12D/+^;*Med23*^+/+^ mice and *Kras*^G12D/+^;*Med23*^f/f^ mice (*n* = 3 per group). **f**, **g** Total RNA was extracted from lung tumors from both groups at 20 weeks post Adeno Cre infection and then the mRNA levels of MAPK pathway-related genes were analyzed by qRT‒PCR (*n* = 7 per group). **h** Total protein was extracted from lung tumors from both groups at 25 weeks post-infection and then the protein levels of MAPK pathway- related genes in lung tumors were analyzed by Western blotting using the indicated antibodies. β-Actin was used as a loading control. *n* = 3 mice per group, and two separate tumors were taken from each mice for Western blot analysis. **i** Quantification of p-Erk (by the gray degree value in H) with normalization to Erk. **j** Lung sections from *Kras*^G12D/+^;*Med23*^+/+^ mice and *Kras*^G12D/+^;*Med23*^f/f^ mice at 16 weeks post Adeno Cre infection were stained for p-Erk, and representative views are shown. Data are presented as the means ± SEMs. *P < 0.05, **P < 0.01, ***P < 0.001.

To explore the cellular mechanisms by which *Med23* deficiency promotes lung tumorigenesis, we analyzed several biological processes associated with tumor formation. We first checked a series of time points of tumor formation after Adeno Cre treatment. At 2 weeks post induction, both groups showed no obvious lesions throughout the whole lungs (Fig. 3a). However, by 8 weeks post induction, there was considerable atypical adenomatous hyperplasia (AAH) and some large adenomas in *Kras*^G12D/+^;*Med23*^f/f^ mice, compared to only a few AAHs in the control group (Fig. 3a). Moreover, by 12 weeks and 16 weeks post Adeno Cre inhalation, the *Kras*^G12D/+^;*Med23*^f/f^ mice clearly formed more and lager tumors, compared to less and smaller tumors in the *Kras*^G12D/+^;*Med23*^+/+^mice (Fig. 3a, b; Fig. S2A, B and C). These data suggest that loss of *Med23* combined with *Kras*-activation unexpectedly leads to enhanced lung tumors progression.

We then further analyzed the proliferation of tumor cells by Ki67 staining. Quantification of Ki67-positive cells demonstrated a statistically significant increase in tumor cell proliferation in the *Kras*^G12D/+^;*Med23*^f/f^ group compared with the control group, which explains the larger tumors observed in *Kras*^G12D/+^;*Med23*^f/f^ mice (Fig. 3c, d). We also compared angiogenesis and DNA damage between the two groups. Quantification of endomucin-positive signals showed no significant difference in angiogenesis (Fig. 3c, e). Both groups showed only occasional γ–H2AX-positive cells, suggesting no difference in DNA damage related to *Med23* deletion (Fig. 3c). Overall, *Med23* deletion appears to enhance the *Kras*-driven tumorigenesis via effects on tumor progression and cell proliferation.

### *Med23* deficiency increases MAPK signaling in *Kras*-driven lung tumors

A recent study demonstrated that a relatively small degree of MAPK signal amplification significantly increases the frequency of *Kras*^G12D^-driven tumor progression and cell proliferation in tumors [23]. Since MED23 is a target of MAPK signaling [21, 22], we investigated whether *Med23* deficiency alters the MAPK pathway. We first evaluated the expression of *Med23* downstream target genes in both groups. Similar to previous study [19], deletion of *Med23* significantly decreased the mRNA expression levels of *Egr1* (Early growth response 1) and its target gene *Nab2* (NGFI-A-binding protein 2) (Fig. 3f, g), suggesting that the functional interaction between MED23 and ELK1 likely functions in *Kras*-induced lung tumors in vivo. In addition, the mRNA levels of *Egr2*, and *Fos* were also decreased in the *Kras*^G12D/+^;*Med23*^f/f^ group compared with the control group, although they were not statistically significant. But the expression of *Jun* seemed not to be changed between the two groups (Fig. 3f). We also examined the mRNA levels of some other MAPK signaling-related genes, such as *Dusp1*, *Dusp6* and *Dusp10*. Among these genes, only *Dusp6* exhibited an approximately 2-fold decrease in the *Kras*^G12D/+^;*Med23*^f/f^ group compared with the control group (Fig. 3g). *Dusp6*, a dual-specificity protein phosphatase, can negatively regulate the MAPK pathway by dephosphorylating p-Erk. We then directly analyzed the p-Erk levels by Western blotting. The results showed a clear increase in the phosphorylated Erk1/2 level after *Med23* deletion, but no changes for the total Erk1/2 level or the protein levels of other MAPK pathway- related genes, such as *Ets1*, *Sprouty2*, *Src* and *p-Akt* (Fig. 3h, i). Immunostaining also verified the enhanced p-Erk level in *Med23*-deficiency lung tumors tissues (Fig. 3j). Overall, the increased phosphorylation of Erk was consistent with the decreased *Dusp6* expression in *Kras*^G12D/+^;*Med23*^f/f^ mice, in which the greatly reduced *Egr1* level indicated the requirement for MED23 in transducing *Kras* signaling. These results indicated that *Med23* deficiency increased MAPK signaling, which was consistent with the increased proliferation and tumorigenesis in the *Kras*-driven lung cancer observed in vivo.

### *Med23* deletion alters the tumor microenvironment by affecting the recruitment of immune cells

**Fig. 4:**
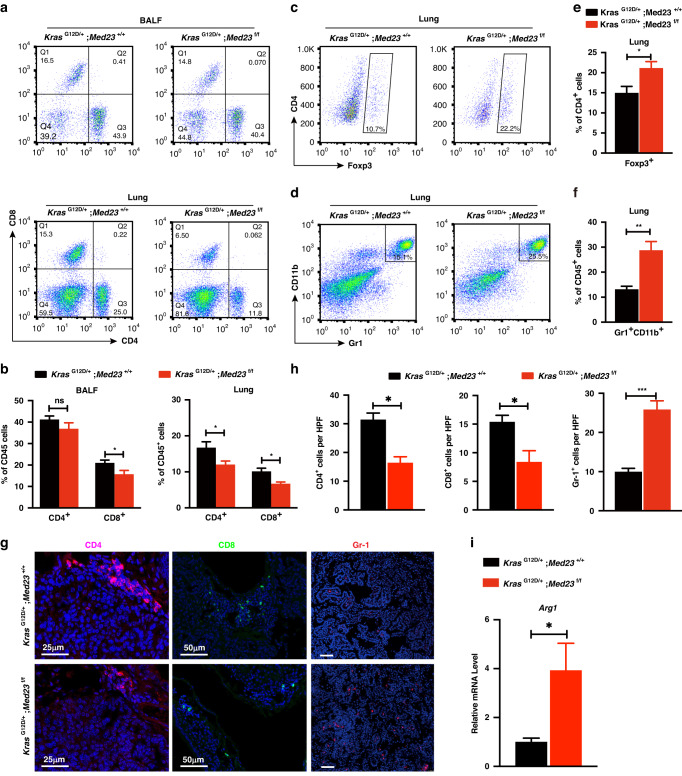
Immune cell infiltration was impaired in *Med23*-deleted mice. Flow cytometric quantification of CD4^+^ and CD8^+^ T cells (in total CD45^+^ T cells) in the BALF and lungs of *Kras*^G12D/+^;*Med23*^+/+^ mice and *Kras*^G12D/+^;*Med23*^f/f^ mice. Representative contour plots (**a**) and quantification (**b**) are shown (*Kras*^G12D/+^;*Med23*^+/+^, n = 8; *Kras*^G12D/+^;*Med23*^f/f^, *n* = 7). **c** Flow cytometric quantification of Foxp3^+^ T cells (in total CD4^+^ T cells) in the lungs for both groups. Representative contour plots are shown (*n* = 8 per group). **d** Flow cytometric quantification of MDSCs in the lungs for both groups. Representative contour plots are shown (*Kras*^G12D/+^;*Med23*^+/+^, *n* = 9; *Kras*^G12D/+^;*Med23*^f/f^, *n* = 8). **e** Quantification of Foxp3^+^ T cells (in total CD4^+^ T cells) in the lungs for both groups (*n* = 8 per group). **f** Quantification of MDSCs in the lungs for both groups (*Kras*^G12D/+^;*Med23*^+/+^, *n* = 9; *Kras*^G12D/+^;*Med23*^f/f^, *n* = 8). **g** Representative views of CD4, CD8 and Gr-1 staining in lung sections from *Kras*^G12D/+^;*Med23*^+/+^ mice and *Kras*^G12D/+^;*Med23*^f/f^ mice at 16 weeks post Adeno Cre infection (*n* = 4 per group). **h** Quantification of CD4, CD8 and Gr-1 positive cells in lung sections from *Kras*^G12D/+^;*Med23*^+/+^ mice and *Kras*^G12D/+^;*Med23*^f/f^ mice at 16 weeks post Adeno Cre infection (*n* = 4 per group). **i** qRT‒PCR analysis of *Arg1* expression in lungs for both groups at 20 weeks post Adeno Cre infection (*n* = 7 per group). Data are presented as the means ± SEMs. *P < 0.05, **P < 0.01, ***P < 0.001.

Since *Med23* deletion exerts opposite effects on lung tumorigenesis in vitro and in vivo, we speculated that host factors, such as the tumor microenvironment, may contribute to the apparent discrepancy between the in vitro and in vivo phenotypes of *Med23* deletion. The tumor microenvironment consists of various nontumor cells present in the tumor, among which immune cells play an important role [24]. Therefore, we examined immune cells in both groups. We first examined CD4^+^ T cells and CD8^+^ T cells, two types of lymphocytes closely associated with tumor progression, in the bronchoalveolar lavage fluid (BALF) by flow cytometric analysis. The results showed a significantly lower percentage of CD8^+^ T cells and a tendency toward a lower percentage of CD4^+^ T cells in the CD45^+^ leukocyte population in *Kras*^G12D/+^;*Med23*^f/f^ mice than in that in *Kras*^G12D/+^;*Med23*^+/+^ mice (Fig. 4a, b). Further, we digested the lungs and directly analyzed the CD4^+^ T cells and CD8^+^ T cells within the lung tissues, the result also confirmed the reduced percentages of CD4^+^ T cells and CD8^+^ T cells in *Kras*^G12D/+^;*Med23*^f/f^ mice compared with control mice (Fig. 4a, b). Moreover, immunohistochemical analysis also verified the decreased number of CD4^+^ T cells and CD8^+^ T cells in *Kras*^G12D/+^;*Med23*^f/f^ mice (Fig. 4g, h).

Regulatory T (Treg) cells are immunosuppressive cells that are defined as Foxp3^+^ cells in mice, and these cells have been shown to promote tumor progression and metastasis through their suppressive functions. We then evaluated Treg cells in the lungs by flow cytometric analysis and observed a significant increase in the percentage of Foxp3^+^ cells in the CD4^+^ population in *Kras*^G12D/+^;*Med23*^f/f^ mice compared with control mice (Fig. 4c, e). These data suggested that lung tumors with *Med23*-deletion associate with the decreased tumor-inhibiting T cells (CD4^+^ T cells and CD8^+^ T cells) and an increase in tumor-promoting T cell (Foxp3^+^ Treg), which may constitute an immune microenvironment promoting the tumorigenesis in *Med23*-deficient mice.

To further understand how T cell populations are changed in the tumor microenvironment, we next examined myeloid-derived suppressor cells (MDSCs), which are a heterogeneous group of myeloid cells broadly defined as CD11b^+^ Gr1^+^ cells in mice. A recent study showed that MDSCs can promote lung tumorigenesis by directly suppressing CD4^+^ T cells and CD8^+^ T cells [23]. We evaluated MDSCs in the lungs by flow cytometric analysis. The percentage of CD11b^+^ Gr1^+^ cells was significantly increased in *Kras*^G12D/+^;*Med23*^f/f^ mice compared with *Kras*^G12D/+^;*Med23*^+/+^ mice (Fig. 4d, f). Immunohistochemical analysis also verified the increase in intra-tumoral Gr-1^+^ cells in *Kras*^G12D/+^;*Med23*^f/f^ mice (Fig. 4g, h). MDSCs can also inhibit effector T-cell responses by supporting Treg cells [25], and indeed, we found that the number of Treg cells was higher in mice with *Med23* deleted (Fig. 4e). Interestingly, we found the mRNA level of *Arg1* (Arginase 1) was significantly increased in mice with *Med23* deletion (Fig. 4i), which is consistent with a report that *Arg1* is secreted by MDSCs to suppress CD8^+^ T-lymphocyte function [26].

Overall, these results suggested that *Med23* deficiency may alter the immune cell recruitment to the tumor microenvironment, enabling the *Med23*-deficient tumors to escape immune surveillance and elimination.

### Immune response related genes were downregulated after *Med23* deletion

**Fig. 5:**
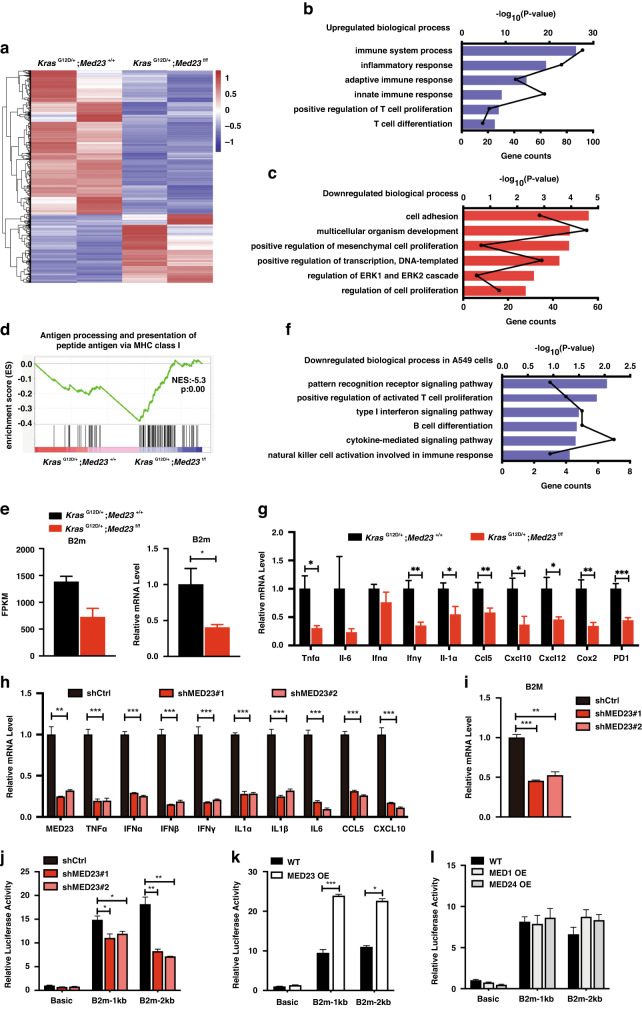
Inflammation-related genes were downregulated in *Med23-*deleted mice. **a** Heatmap analysis of differentially expressed genes between *Kras*^G12D/+^;*Med23*^+/+^ mice and *Kras*^G12D/+^;*Med23*^f/f^ mice at 20 weeks post-infection. GO analysis of downregulated (**b**) and upregulated (**c**) genes (fold change > 2) in *Kras*^G12D/+^;*Med23*^f/f^ mice. The black line indicates the gene counts and the blue bars indicate the statistics. **d** GSEA plot showing enrichment of antigen processing and presentation of a peptide antigen via MHC class I in *Kras*^G12D/+^;*Med23*^+/+^ mice. **e** The mRNA levels of *B2m* were confirmed by qRT‒PCR (*n* = 7 per group). **f** GO analysis of downregulated genes (fold change > 2) in *MED23*-knockdown A549 cells. The black line indicates the gene counts, and the blue bars indicate the statistics. **g** qRT‒PCR analysis of inflammation-related genes in lung tumors for both groups at 20 weeks post-Adeno Cre infection (*n* = 7 per group). **h** qRT‒PCR analysis of inflammation-related genes in A549 cells (*n* = 3 per group). **i** The mRNA level of *B2M* in A549 cells was analyzed by qRT‒PCR (*n* = 3 per group). The effect of *Med23* knockdown (**j**) and *Med23* overexpression (**k**) identified with a *B2m* promoter-luciferase reporter assay is shown (*n* = 3 per group). **l** The effect of overexpression of *Med1* and *Med24* on the *B2m* promoter-luciferase reporter assay is shown (*n* = 3 per group). Data are presented as the means ± SEMs. * *P* < 0.05, ** *P* < 0.01, *** *P* < 0.001.

To understand the molecular basis of the altered immune microenvironment by *Med23* deficiency, RNA-seq analysis of lung tumors with or without Med23 were performed. We found 1524 genes downregulated more than 2-fold and 728 genes upregulated more than 2-fold in the *Kras*^G12D/+^;*Med23*^f/f^ mice relative to the control mice (Fig. 5a). Gene Ontology (GO) analysis of the downregulated genes identified biological processes important in the immune response and T-cell proliferation, while the upregulated genes were related to processes involved in cell adhesion and cell proliferation (Fig. 5b, c). qPT-PCR further confirmed that the mRNA levels of most cytokines including *Tnfα*, *Ifnγ*, *Il-1α*, *Ccl5* and *Cxcl10* were markedly decreased in the Med23-deficient tumors (Fig. 5g), which may lead to fewer CD4^+^ and CD8^+^ T cells recruitment. These data are consistent with the phenotype of a compromised immune microenvironment and the enhanced tumor growth of the *Kras*^G12D/+^;*Med23*^f/f^ mice.

To explore what caused these immune response change in *Med23*-deleted mice, we performed gene set enrichment analysis (GSEA) and revealed that *Med23*-downregulated genes were enriched in antigen processing and presentation of peptide antigens via MHC class I (Fig. 5d). MHC-I molecules are required for cytotoxic lymphocyte (CTL) cell function. A previous study found that downregulation of MHC class I molecule expression could allow tumor cells to evade the host immune response, thereby rendering any endogenous or therapeutic antitumor T-cell responses ineffective [27]. Notably, one such gene, *B2m*, which is an important component of MHC class I molecules [28], was found to be downregulated in *Kras*^G12D/+^;*Med23*^f/f^ mice compared to *Kras*^G12D/+^;*Med23*^+/+^ mice (Fig. 5e). These results suggested that *Med23* deficiency might support immune evasion by downregulating the expression of immune response-related genes to promote lung tumorigenesis.

Next, we asked whether this negative regulation of immune response-related genes by *Med23* deletion occurs in a cell autonomous manner. To address this, we reanalyzed our previous microarray data using lung cancer cell line [19]. To our surprise, GO analysis of the genes downregulated in retrovirally-mediated *MED23*-knockdown A549 cells showed that these genes were related to processes involved in T-cell proliferation and cytokine-mediated signaling pathways, consistent with what we observed in the mouse model (Fig. 5f). We further examined the mRNA levels of inflammatory mediators in A549 cells by qRT-PCR, and found that the levels of most inflammatory mediators were obviously decreased in retrovirally-mediated *MED23*-knockdown cells relative to the corresponding control cells (Fig. 5h). Additionally, the mRNA level of *B2M* was decreased in *MED23*-knockdown A549 cells (Fig. 5i) and flow-based quantification of cell surface MHC-I in A549 cells showed that MHC-I expression was also decreased by *MED23* knockdown (Fig. S3A). These results suggested that cancer cells lacking *MED23* may exhibit diminished ability in recruiting CD8 and CD4 T lymphocytes, which possibly contributing to enhanced tumor growth in vivo.

We also examined how *MED23*-deficieny might regulate *B2M* expression in vitro. Utilizing luciferase reporter assays, we found that *MED23* knockdown reduced *B2M* promoter reporter activity, while *MED23* overexpression enhanced its activity (Fig. 5j, k). In contrast, overexpression of other Mediator subunits, *MED1* and *MED24*, did not change the activity of the *B2M* promoter (Fig. 5l). These results underscored the specific effect of MED23 in supporting *B2M* expression.

Lastly, since *Med23* deficiency in mice is apparently important for the growth of non-small cell lung cancer, we examined whether there were also lung cancer patients with *Med23* downregulation or deletion. Indeed, we found that approximately 2.1% of lung adenocarcinoma patient samples in the TCGA database had mutations in *MED23* utilizing relevant website analysis [29, 30] (Fig. S4A), and most of the mutations in *MED23* led to a reduction in the copy number of *MED23* (Fig. S4B, C). Furthermore, we reanalyzed a cDNA array derived from 24 lung adenocarcinoma patient samples reported in our previous study [19] to determine the mRNA levels of *MED23* in tumor and adjacent tissues. While there was increased *MED23* expression in 7 out of 24 patient samples as previously found [19], we noticed that *MED23* was downregulated in the tumors of 4 out of 24 patients (16.7%) (Fig. S4D). Finally, we found that patients with low MED23 expression exhibited significantly shorter survival times using the Kaplan‒Meier Plotter website [31] (Fig. S4E). Collectively, these results indicate that there is indeed a small fraction of patients with low MED23 expression in the clinic and that our research may provide a new classification for lung cancer patients.

## Discussion

To elucidate the role of MED23 in lung tumorigenesis in vivo, we used the well-characterized *Kras*^G12D^ lung tumor mouse model and demonstrated that *Med23* deletion in vivo showed enhanced lung tumorigenesis, possibly by increasing the proliferation of lung tumor cells and reprogramming the immune microenvironment to promote lung tumor growth.

Our previous study found that MED23 could selectively support the cell growth and tumorigenesis of lung cancer cell lines with mutant *KRAS* in vitro [19]. However, in contrast, in the present study, we found that *Med23* deletion in vivo enhanced *Kras*-induced lung tumorigenesis. To understand this paradox, we hypothesized that the tumor microenvironment may contribute to the difference between the in vitro and in vivo phenotypes of *Med23* deficiency. The tumor microenvironment is dynamic during tumorigenesis, within it, immune cells are considered to be key components of the antitumor immune response and immune escape. On the one hand, CD8^+^ T cells and NK cells can directly kill tumor cells and inhibit tumorigenesis; on the other hand, some suppressive immune cells (MDSCs, Treg cells, etc.) can inhibit T-cell responses and promote matrix degradation, tumor cell proliferation, metastasis and blood vessel formation to support tumor development [32]. MED23 has been reported to control T-cell activation, and *Med23* deletion in T cells enhances antitumor T-cell function [33]. Therefore, the role of *Med23* in the tumor-directed immune response is complex and might be context dependent. In this study, we observed that the percentages of CD4^+^ T cells and CD8^+^ T cells in the lungs were reduced, while the numbers of MDSCs and Treg cells were increased in *Kras*^G12D/+^;*Med23*^f/f^ mice compared with control mice (Fig. 4). We also found that the expression of most cytokines and chemokines was decreased after *Med23* deletion in a seemingly cell-autonomous manner.

During the process of tumorigenesis, the immune system is able to recognize the nonself antigens represented by cancer cells and eventually eliminate the cancer cells. However, tumor cells can avoid being recognized by immune cells by changing their own surface antigens or reducing antigen expression to escape attack by immune cells and continue to proliferate. In this study, GSEA showed that antigen processing and presentation of peptide antigens were impaired in *Kras*^G12D/+^;*Med23*^f/f^ mice. Interestingly, we found that the expression of a gene called *B2m* (Beta-2-Microglobulin) was significantly reduced in the tumors of *Kras*^G12D/+^;*Med23*^f/f^ mice. *B2m* is the invariant light chain of the major histocompatibility complex class I (MHC-I) molecule and is important for maintaining the structural stability of the MCH-I complex [34]. The MHC-I complex expressed on the surface of tumor cells can recognize, process and present tumor neoantigens and thus activate CD8^+^ T lymphocytes. Inactivation of the MHC-I complex has been reported in many human cancers, and *B2m* mutations have been found in various tumor types, including lung cancers [35, 36]. Although the in vivo and in vitro phenotypes of *Med23* deletion are different, we found that the mRNA level of *B2m* was decreased after *MED23* knockdown in A549 cells. Therefore, the decrease in the *B2m* expression level caused by *Med23* deletion might at least partially account for the compromised MHC-I complex formation and enhanced tumorigenesis observed. We thus propose the following model: MED23 may control immunogenicity, as *MED23* deletion causes low levels of the MHC-I complex accompanied by low levels of CD8^+^ T lymphocytes, cytokines, chemokines and PD1, which formed a noninflamed (or cold) tumor microenvironment to promote tumorigenesis. The absence of *Med23* impairs the recruitment of immune cells, possibly contributing to an immunosuppressive microenvironment and ultimately promoting lung tumorigenesis. However, it is technically challenging for us to investigate the stimulation of CD4^+^ and CD8^+^ T cells in vivo and its direct effect on tumor burden in *Med23* knockout mice and the detailed mechanism needs to be further studied. Collectively, the in vivo lung tumorigenesis model captures a broader biological scenario, and the interactions with the immune system may contribute to the observed differences compared with in vitro.

Utilizing tissue microarrays to analyze 80 clinical samples of lung cancer, we previously found that nearly 85% of the lung cancer samples had significantly higher expression of MED23 than did normal tissue samples [19]. Interestingly, when reanalyzed the cDNA array data we previously published, we found that not all the patients had high MED23 expression in their tumor tissues and that a few patients (4/24) showed lower MED23 expression in their tumor tissues than in adjacent tissue [19]. We then further analyzed the lung cancer samples collected in the TCGA database through relevant websites and found that there were indeed some patients with relatively low MED23 expression or even MED23 deletion. These clinical data suggest that MED23 expression probably needs to be balanced in cells. Too much or too little of MED23 may dysregulate lung homeostasis. Here, we found that loss of *Med23* affected the infiltration of immune cells and the tumor microenvironment, which implied immunotherapy might have better efficacy in patients with low MED23 expression.

In summary, this study reveals that deficiency in the Mediator component MED23 may aggravate *Kras*-induced lung tumorigenesis in vivo by reprogramming the tumor microenvironment. These findings provide new understandings and potential new strategies for the stratification and treatment of lung cancer patients with *KRAS* mutations. In the future, drugs targeting MED23 combined with immunotherapy may specifically benefit patients with lung cancer with relatively low MED23 expression.

## Materials and methods

### Ethics statement

All animal experiments were conducted using mice bred at and maintained in our animal facility according to the guidelines of the Institutional Animal Care and Use Committee of CAS Center for Excellence in Molecular Cell Science (IACUC-PR01).

### Mice and tumor models

*LSL-Kras* ^G12D/+^ mice and *Spc*-*Cre*^ERT2^ mice were generously provided by Hongbin Ji (CAS Center for Excellence in Molecular Cell Science, China). We crossed the *LSL-Kras*^G12D/+^ mice with *Med23*^f/f^ mice to generate *LSL-Kras*^G12D/+^;*Med23*^+/+^ mice and *LSL-Kras*^G12D/+^;*Med23*^f/f^ mice [37]. Both male and female mice were used for experiment. To achieve activation of *Kras*^G12D^ with or without simultaneous deletion of *Med23* in lung epithelial cells, 6- to 8-week-old mice were treated with 2 × 10^6^ PFU of Adeno Cre virus via nasal inhalation as described previously [38]. In addition, to generate mice with *Med23* deletion specifically in type II alveolar epithelial cells, *Med23* ^f/f^ mice were crossed with *Spc*-*Cre*^ERT2^ mice [39]. To induce Cre activity, 8-week-old mice were intraperitoneally injected with 100 μl of tamoxifen (100 mg/ml) for five consecutive days. All mice were sacrificed for gross inspection and histopathological examination. Lung tumors were dissected for molecular analysis.

### Histological analysis

H&E staining and immunohistochemistry were performed on formalin-fixed paraffin -embedded lung sections as described previously [19]. The following antibodies were used for staining: anti-SFTPC (Millipore, AB3786, RRID: AB_91588), anti-TTF1 (RabMAb, EPR8190), anti-Ki67 (Novocastra, NCL-Ki67p), anti-Endomucin (Thermo Fisher Scientific, 14-5851-81, RRID: AB_891529), anti-γ-H2AX (Cell Signaling Technology, 9718), anti-p-Erk1/2 (Cell Signaling Technology, 4370), anti-CD4 (Abcam, ab183685), anti-CD8 (Cell Signaling Technology, 98941).

For Gr-1 immunofluorescence staining, lung cryosections were placed at room temperature for 1–2 h, fixed with precooled acetone for 10 min and washed with phosphate-buffered saline (PBS) three times. After immersion in 10% normal goat serum, the sections were sequentially incubated with a PE-conjugated anti-mouse Gr-1 antibody (BioLegend, 108407) overnight at 4 °C. The sections were washed with PBS three times, and nuclei were stained with diluted DAPI (0.5 μg/ml) for 5 min. Finally, the sections were mounted with fluorescence mounting medium (DAKO).

For quantification, the tumor number was counted under microscope, and tumor area was quantified using ImageJ software by measuring pixel units. The number of Ki67, CD4, CD8 and Gr-1 cells were evaluated by counting Ki67, CD4, CD8 or Gr-1 positive staining at high-power field for 30 fields for each group. The quantification was performed with double blind.

### Assessment of survival rate in the tumor models

Eight-week-old *Kras*^G12D/+^;*Med23*^+/+^ mice and *Kras*^G12D/+^;*Med23*^f/f^ mice were treated with 1.2 × 10^7^ PFU of Adeno Cre virus via nasal inhalation. They were maintained in a specific pathogen-free facility and monitored every week throughout the indicated time period to calculate the survival rate. For ethical reasons and humane considerations, the study being stopped before control animals died naturally.

### Quantitative real-time PCR (qRT‒PCR)

Total RNA was extracted from cells or lung tumors from *Kras*^G12D/+^;*Med23*^+/+^ mice and *Kras*^G12D/+^;*Med23*^f/f^ mice post 20 weeks Adeno Cre infection using TRIzol (Thermo, 15596018). Reverse transcription and qRT‒PCR were performed as described previously [18]. Mouse gene expression levels were normalized to 18S RNA expression levels, while human gene expression levels were normalized to *β-ACTIN* expression levels. The primers are listed in Supplementary Table 1 in Supplementary Materials.

### Western blotting

Western blotting was carried out as described previously [37]. The following primary antibodies were used for western blotting: anti-MED23 (BD, 550492), anti-MED16 (Bethyl, A303-668A, RRID: AB_11205632), anti-MED24 (Bethyl, A301-472A, RRID: AB_999675), anti-phosphorylated-Erk1/2 (Thr202/Tyr204) (Santa Cruz, sc-16982), anti-Erk (K-23) (Santa Cruz Biotechnology, sc-94-G, RRID: AB_631456), anti-phosphorylated-Akt (S473) (Cell signaling, 9271), anti-Akt (Cell signaling Technology, 9272, RRID: AB_329827), anti-Ets1 (Santa Cruz, sc-350x), anti-Sprouty2 (Santa Cruz, sc-30049), anti-Src (Cell signaling Technology, 2108s, RRID: AB_331137) and β-Actin (Sigma, A5441).

### Bronchoalveolar lavage fluid (BALF) collection

The thorax of mice was cut until the trachea was exposed, and then 0.8 ml of precooled PBS was injected into the trachea twice through a vein-detained needle to wash the lungs. The first collection of BALF was stored at −80 °C for further analysis. The second collection of BALF was centrifuged at 6000 rpm for 1 min at 4 °C. The cell pellet was resuspended in FACS buffer (PBS with 2% FBS) for flow cytometric analysis.

### Isolation of mouse lung cells

Lungs were perfused with 20 ml of cold PBS after collecting the BALF, and the whole lungs were resected. One left lobe was used for histological analysis, and five right lobes were used for flow cytometric analysis. Whole right lobes were washed with PBS twice, diced into small pieces and digested with 5 ml of collagenase-containing buffer (250 U/ml collagenase type IV, 50 μg/ml DNase I and 10% FBS in RPMI-1640 medium) at 37 °C for 1–1.5 h. Digestion was stopped by adding the same volume of medium, and the digest tissue samples were filtered through a 100-μm cell strainer. The cells were collected by centrifugation at 1500 rpm for 20 min at 4 °C. The cell pellets were treated with red blood cell lysis buffer for 4 min and filtered through a 40-μm cell strainer. Then, the cells were collected by centrifugation at 1500 rpm for 20 min at 4 °C. The cell pellets were resuspended in FACS buffer and centrifuged at 6000 rpm for 1 min at 4 °C. Finally, the cell pellets were resuspended in FACS buffer and counted for flow cytometric analysis.

### Flow cytometric analysis of immune cells

We washed cells with FACS buffer and collected the cell pellets by centrifugation at 6000 rpm for 1 min at 4 °C. The cell pellets were resuspended in 100 μl of FACS buffer, treated with 1 μl of Fc block and incubated on ice for 15 min. For detection of CD4- and CD8-positive cells in the BALF and lungs, cells were incubated on ice for 40 min with the following antibodies: FITC-conjugated anti-mouse CD45 (BioLegend, 103108, RRID: AB_312973), PerCP-Cyanine5.5-conjugated anti-mouse CD4 (eBioscience, 45-0042-82) and APC-eFluor 780-conjugated anti-mouse CD8a (Thermo Fisher Scientific, 45-0081-82, RRID: AB_1107004). For the detection of MDSCs in the lungs, cells were incubated with APC-conjugated anti-mouse/human CD11b (BioLegend, 101212) and PE-conjugated anti-mouse Gr1 (BioLegend, 108407). Then, the cells were washed with PBS twice and centrifuged at 6000 rpm for 1 min at 4 °C. The cell pellets were resuspended in 1 ml of PBS and stained with Fixable Viability Dye eFluor 455UV on ice for 30 min. In addition, intracellular Foxp3 staining was performed with PE-conjugated anti-mouse/rat Foxp3 (eBioscience, 12-5773-82) and Foxp3 Fixation/Permeabilization Buffer according to the manufacturer’s instructions. After washing, FACS was performed with an LSRII instrument (BD Biosciences, RRID: SCR_013311), and the data were analyzed with FlowJo (RRID: SCR_008520) software (BD Biosciences).

### RNA-seq

Total RNA was extracted from the lung tumors of *Kras*^G12D/+^;*Med23*^+/+^ mice and *Kras*^G12D/+^;*Med23*^f/f^ mice post 20 weeks Adeno Cre infection using TRIzol (Thermo Fisher Scientific, 15596018). RNA integrity was evaluated using an Agilent Bioanalyzer. cDNA libraries were prepared as described previously and subjected to 75-bases sequencing on a Genome Analyzer IIx (Illumina) according to Illumina’s standard protocols. Single-end reads were aligned to the mouse genome mm9 using Tophat2 [40]. The differential gene expression between groups was analyzed by Cuffdiff [40]. Genes with |fold change | >2 were selected for further analysis. Gene Ontology (GO) analysis of differentially expressed genes was performed by DAVID v6.8. Gene set enrichment analyses (GSEA) were performed with the GSEA platform using the pre-ranked list of differentially expressed genes. The RNA-seq data in this study have been deposited in the Gene Expression Omnibus (GEO) (RRID: SCR_005012) database. The A549 cell microarray data used for GO analysis are attached in Supplementary Table 2 in  Supplementary Materials.

### Luciferase reporter assay

A549 cells were periodically tested mycoplasma negative and seeded into a 12-well plate at 1 × 10^5^ cells per well overnight. These cells were then transfected by Lipofectamine 2000 (Invitrogen, 11668019) with a luciferase reporter plasmid and pRL-TK (Promega) along with various expression constructs, as indicated. All wells were supplemented with control empty expression vector plasmids to keep the total amount of DNA constant. The cells were harvested and subjected to dual-luciferase reporter assays after 36–48 h of transfection according to the manufacturer’s protocol (Promega). A549 cells were further validated by STR profiling at the end of the study to ensure cell identity.

### Statistical analysis

All data are presented as the means ± SEMs. Differences between groups were compared with a two-tailed unpaired Student’s *t* test or the Mann‒Whitney test using GraphPad Prism (Version 8.0) (RRID: SCR_002798) before compared the variance. Survival was analyzed by the Kaplan‒Meier method and compared with the log-rank test. When *P* values < 0.05, differences were considered statistically significant.

### Supplementary information

Supplementary Figure legends

Supplementary Figure 1

Supplementary Figure 2

Supplementary Figure 3

Supplementary Figure 4

Supplementary Table 1

Supplementary Table 2

## Data Availability

All relevant data are available from the authors upon request.
