# Supplementary material for: Med23 deficiency reprograms the tumor microenvironment to promote lung tumorigenesis
Source: Br J Cancer. 2024 Jan 9;130(5):716–27. doi: 10.1038/s41416-023-02556-9 (PMC10912217; doi:10.1038/s41416-023-02556-9)
Supplement: Supplementary file 1 — Supplementary Figure legends [file 41416_2023_2556_MOESM1_ESM.docx]

**Supplementary Figure 1. Analysis of lung tumors in *Kras*^G12D/+^;*Med23*^+/+^ mice and *Kras*^G12D/+^;*Med23*^f/f^ mice.** (A) Representative views of H&E staining of lung sections from *Med23*^f/f^ mice at 25 weeks post Adeno Cre infection. (B) H&E staining of lung sections from *Kras*^G12D/+^;*Med23*^+/+^ mice and *Kras*^G12D/+^;*Med23*^f/f^ mice. (C) Representative views of lung sections from *Kras*^G12D/+^;*Med23*^+/+^ mice, *Kras*^G12D/+^;*Med23*^f/+^ mice and *Kras*^G12D/+^;*Med23*^f/f^ mice. (D) Quantification of the tumor number and tumor size of *Kras*^G12D/+^;*Med23*^+/+^ mice, *Kras*^G12D/+^;*Med23*^f/+^ mice and *Kras*^G12D/+^;*Med23*^f/f^ mice (*Kras*^G12D/+^;*Med23*^+/+^, n=7; *Kras*^G12D/+^;*Med23*^f/+^ mice, n=7; *Kras*^G12D/+^;*Med23*^f/f^, n=8). Data are presented as the means ± SEMs. ** P < 0.01, *** P < 0.001.

**Supplementary Figure 2. *Med23* deficiency promotes tumor progression.** (A-C) Quantification of the tumor number and tumor size of *Kras*^G12D/+^;*Med23*^+/+^ mice and *Kras*^G12D/+^;*Med23*^f/f^ mice at the indicated times (n=3 per group).

**Supplementary Figure 3. MED23 knockdown decreases MHC-I expression in A549 cells.** (A) Flow cytometric analysis of MHC-I expression in A549 cells with or without *MED23* knockdown.

**Supplementary Figure 4. Expression pattern of MED23 in clinical lung cancer patients.** (A-C) The expression of and detailed mutations in MED23 in clinical lung cancer samples on the cBioPortal website. (D) qRT‒PCR analysis of MED23 expression in 24 lung cancer patients. The blue box denotes 4 patients with low MED23 expression. (E) Kaplan‒Meier survival curves for MED23-high and MED23-low lung cancer patients (MED23-high, n=357; MED23-low, n=362).
